# Large Language Model Automated Extraction of Clinical Signs and Symptoms From Emergency Department Reports for Machine Learning Prediction Models: Development and Validation Study

**DOI:** 10.2196/81500

**Published:** 2026-04-30

**Authors:** Anoeska Schipper, Peter Belgers, Rory David O'Connor, Lieke van de Wouw, Luc Builtjes, Joeran S Bosma, Ron Kusters, Steef Kurstjens, Matthieu Rutten, Bram van Ginneken

**Affiliations:** 1 Diagnostic Image Analysis Group, Medical Imaging Department, Radboud University Medical Center, Geert Grooteplein Zuid 10, Nijmegen, 6525 GA, The Netherlands, 31 614021323; 2 Laboratory of Clinical Chemistry and Hematology, Jeroen Bosch Ziekenhuis, Den Bosch, The Netherlands; 3 Psychiatry Department, Radboud University Medical Center, Nijmegen, The Netherlands; 4 Emergency Department, Jeroen Bosch Ziekenhuis, Den Bosch, The Netherlands; 5 Department of Health Technology and Services Research, Technical Medical Centre, University of Twente, Enschede, The Netherlands; 6 Laboratory of Clinical Chemistry and Laboratory Medicine, Dicoon BV, Canisius-Wilhelmina Ziekenhuis, Nijmegen, The Netherlands; 7 Medical Imaging Department, Jeroen Bosch Ziekenhuis, Den Bosch, The Netherlands

**Keywords:** large language models, natural language processing, electronic health records, machine learning, predictive modeling, health informatics, emergency medicine

## Abstract

**Background:**

Most clinically relevant information in emergency department (ED) visits is documented in free text, limiting reuse for research and clinical decision support. Despite growing interest in large language model (LLM)–based feature extraction, very few studies have examined it directly on ED reports. Existing work has mainly addressed binary tasks and rarely evaluated their impact on downstream prediction models. Furthermore, evidence for small multilingual LLMs remains limited, especially for underrepresented languages such as Dutch. Locally deployable LLMs could enable automated feature extraction for decision support systems without increasing physician workload.

**Objective:**

We aim to evaluate whether a small open-source LLM (Qwen 2.5:14B) can automatically extract 16 clinical signs and symptoms from ED reports and use these as input for an appendicitis prediction model. LLM performance under minimal and optimized 0-shot prompts was assessed against researcher annotations (reference standard) and physician annotations.

**Methods:**

This retrospective study used 336 ED reports from patients presenting with acute abdominal pain to a Dutch teaching hospital (2016-2023). One hundred reports were randomly selected to develop a minimal and an optimized 0-shot prompt strategy. The remaining 236 reports, reserved for evaluation, were annotated by 2 ED physicians and processed by the LLM to extract 16 signs and symptoms, covering binary, multiclass, and multilabel classification tasks. These features were used as input to the HIVE (History, Intake, Vitals, Examination) appendicitis prediction model. LLM extraction accuracy, sensitivity, and specificity were measured against the researcher’s (reference standard) and physician annotations. The HIVE model’s area under the receiver operating characteristic curve was evaluated using LLM-extracted vs physician-annotated features.

**Results:**

Among 336 ED reports from patients with acute abdominal pain (median age 41, IQR 22‐62 years, 205/336, 61% female), 50% (167/336) had appendicitis. The LLM achieved weighted average accuracies of 0.910 (95% CI (0.018) with minimal prompts and 0.929 (95% CI ±0.016) with optimized prompts, vs 0.961 (95% CI ±0.012) and 0.951 (95% CI ±0.015) for physicians. Corresponding HIVE model area under the receiver operating characteristic curves were 0.871 (95% CI ±0.019) and 0.911 (95% CI ±0.014) with LLM inputs under the minimal and optimized prompts, compared to 0.917 (95% CI ±0.015) and 0.924 (95% CI ±0.018) for physician inputs.

**Conclusions:**

A small locally deployable multilingual LLM can approach physician-level accuracy in extracting structured binary, multiclass, and multilabel clinical data from free-text Dutch ED reports, while preserving patient privacy, interpretability, and statistical transparency for downstream diagnostic modeling.

## Introduction

In the emergency department (ED), clinical information is primarily documented as free-text. While this documentation style aligns with routine clinical practice, it presents challenges for reusing data in research and decision support applications [1-5]. Structured templates in the electronic health record (EHR) system offer a common workaround, but impose extra workload on already time-pressed ED physicians and are rarely adopted in practice [6,7]. Automated feature extraction using natural language processing (NLP) offers a more sustainable alternative. To date, extracting clinical data from ED reports has primarily relied on long short-term memory and BERT (Bidirectional Encoder Representations From Transformers) models, which perform reasonably well but require extensive preprocessing, large manually annotated corpora, and model retraining [8-10].

Generative pretrained large language models (LLMs) present an alternative, as they can be optimized using only prompting, making them more accessible for downstream tasks. While these LLMs have proved effective in extracting diverse features from radiology and pathology reports [11-13], their use in automated feature extraction from ED reports is limited. Unlike radiology or pathology reports, ED reports contain extensive abbreviations, succinct phrasing, and domain-specific terminology, presenting unique challenges to NLP. Recent work has begun to explore LLM-based information extraction in emergency medicine. McMurry et al [14] demonstrated LLM-based identification of binary respiratory symptoms using 0-shot prompting and multicenter validation, while Bejan et al [15] phenotyped symptomatic kidney-stone presentations using a range of prompting and fine-tuning strategies. Gao et al [16] used LLM-generated severity scores, supported by in-context learning and retrieval-augmented generation, to improve early triage predictions. Collectively, recent studies demonstrate the potential of LLM-based feature extraction from ED reports. However, these efforts primarily addressed binary outcomes or limited symptom sets, or did not investigate the downstream impact on diagnostic model performance. Prior work also relied mainly on large LLMs or Chinese bilingual LLMs, leaving open whether smaller multilingual LLMs can perform reliably in underrepresented languages [17]. This is particularly relevant for the Dutch, where the inherent complexity of ED reports is compounded by limited representation in LLM training corpora.

This study addresses these gaps by evaluating whether a small multilingual LLM (Qwen 2.5:14B) can automatically extract binary, multiclass, and multilabel clinical features from Dutch ED reports and provide reliable inputs for a downstream prediction model. Our focus is a clinically relevant use case: acute abdominal pain (AAP). Extracted features are used as input for the HIVE (History, Intake, Vitals, Examination) model, a previously established appendicitis prediction model based on 16 contributing clinical signs and symptoms [18]. These features were originally annotated manually, a process that is labor-intensive and difficult to scale. This study evaluates whether an LLM can automate this extraction and produce comparable inputs by developing and validating two 0-shot prompting strategies. Secondary outcomes included comparison to physician annotations and assessing the appendicitis prediction model using LLM-extracted vs physician-annotated features. We hypothesize that, with appropriate prompting, a small locally run multilingual LLM could achieve near-expert accuracy in feature extraction and maintain comparable predictive models’ performance, supporting the feasibility of a scalable, privacy-preserving workflow for decision support systems at the ED.

## Methods

### Data Collection

Data from 350 patients with AAP were retrospectively collected at Jeroen Bosch Hospital, a Dutch teaching hospital, between July 2016 and January 2023. Patient inclusion criteria and cohort construction are detailed in our previous study [18]. This dataset included 167 appendicitis cases with 169 other AAP presentations. Among other AAP presentations, those suspected of appendicitis were balanced with those having nonspecific or other AAP causes based on initial ED assessments by triage nurses or referring physicians. Cases lacking sufficient medical history or physical examination data or missing more than 70% (n=14) of vital signs were excluded, resulting in 336 cases. Each case contained triage and intake data, vital signs, and the medical history and physical examination sections. Further cohort details are in Supplemental Tables 1A-E of our previous publication [18]. No exclusions were made based on age, comorbidities, medication use, or symptom presentation. Data were extracted and pseudonymized using CTcue (IQVIA Nederland B.V.).

### Study Design

All 336 ED reports were independently annotated by 2 lead researchers to establish the reference standard for 16 clinical signs and symptoms, as previously described [18] (Multimedia Appendix 1). Interrater agreement, assessed on a random sample of 80 reports, demonstrated high reliability, with average Krippendorff α values of 0.93 for binary features and 0.95 for the multiclass feature, and an average Jaccard similarity of 0.76 for multilabel features. The 16 features were derived from our previous HIVE model, in which XGBoost (Extreme Gradient Boosting) and SHAP (Shapley Additive Explanations) analyses were used to identify features contributing more than 95% of the model’s predictive output (Multimedia Appendix 2). Their discriminative performance was previously assessed using the area under the receiver operating characteristic curve (AUROC) to determine the optimal feature set. The final selection comprised 11 medical history and 5 physical examination features, covering 8 binary, 1 multiclass, and 7 multilabel outputs.

LLM-based feature extraction using a 0-shot prompting strategy was chosen over conventional tokenization-based text classification because the goal was to extract clinically meaningful variables and integrate them with structured vital signs and intake information directly available from the EHR. Traditional token-based NLP methods focus on identifying or classifying text spans and cannot reliably capture negation, multilabel outputs, or nuanced clinical context, and typically provide token-level outputs that obscure the contribution of specific clinical findings. This reduces interpretability and alters feature-importance patterns (eg, via SHAP), limiting transparency and alignment with the structured design of the HIVE model.

**Figure 1.**
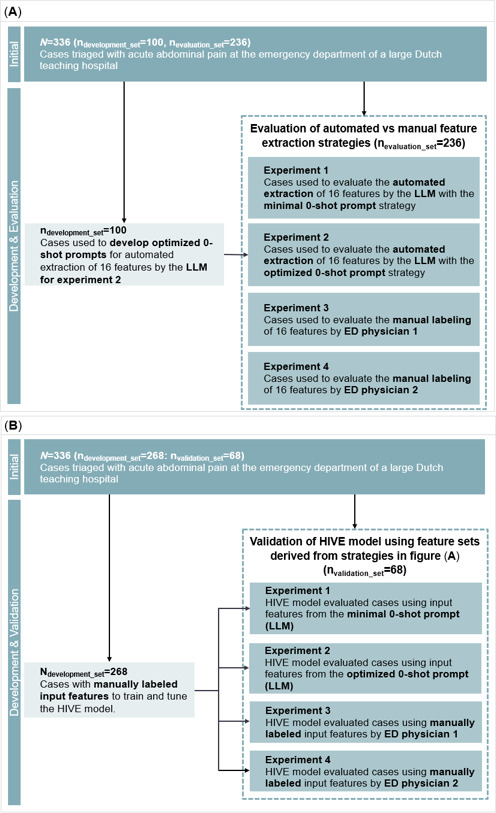
Study design overview comparing feature extraction strategies for the HIVE appendicitis prediction model. (A) Flowchart illustrating the experimental setup for comparing automated (LLM-based) and manual (physician-labeled) feature extraction strategies. All four experiments were conducted on the same set of 236 evaluation cases. (B) Flowchart outlining the HIVE model development and validation process. The model was trained and tuned on a separate, manually labeled development set (n=268), and validated on 68 cases drawn from the same pool of 236 cases used in panel (A). The same trained model was used across all 4 validation experiments; only the feature extraction method varied for the identical set of 68 validation cases. ED: emergency department; HIVE: History, Intake, Vitals, Examination; LLM: large language model; ML: machine learning.

For the present analysis, 100 reports were used to optimize two 0-shot prompting strategies for extracting these features using an LLM. Zero-shot prompting directly instructs the LLM without examples [19]. The remaining 236 reports were reserved to evaluate manual labeling by 2 ED physicians and automated extraction by the LLM (Figure 1A).

These reports were also used for the HIVE appendicitis prediction model. Of the 336 reports, 268 had previously been labeled by the lead researchers and were used for model training and tuning. The remaining 68 reports, drawn from the 236 physician-labeled and LLM-processed reports for evaluation, served as 4 identical validation sets (Figure 1B). This validation set was selected before model development, contained no cases used in LLM prompt construction or hyperparameter tuning, and matched the validation size used in our previous study [18]. This ensured a fully independent and consistent benchmark for assessing the effect of LLM-based feature extraction on downstream model performance. Given the modest dataset size (n=336) and single-center design, this study aimed to evaluate feasibility rather than generalizable performance. The results should therefore be interpreted as proof-of-concept.

### Workflow and Prompting Strategies

**Figure 2.**
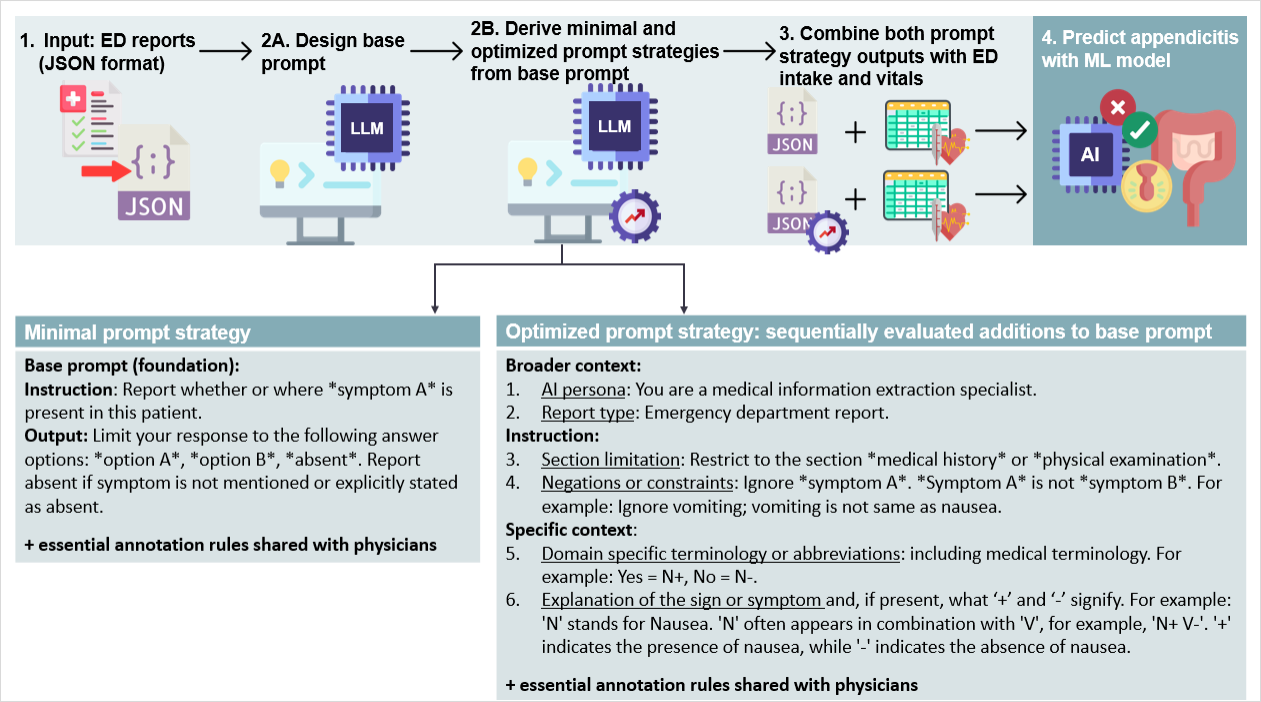
Modular workflow for extracting clinical signs and symptoms using a local LLM, followed by appendicitis prediction. ED reports in JSON format (1) were processed using a base prompt (0-shot) (2A) containing an instruction and predefined output format. From this base, two prompting strategies were derived (2B): a minimal prompt, which included essential clarifications also provided to ED physicians for equal comparison, and an optimized prompt, integrating additional elements that were sequentially evaluated through an ablation study. Feature outputs from both strategies were combined with ED intake and vital-sign data (3) and entered into the HIVE ML model to predict appendicitis (4). AI: artificial intelligence; ED: emergency department; HIVE: History, Intake, Vitals, Examination; LLM: large language model; ML: machine learning.

The primary objective of this study was to evaluate the LLM’s automated extraction of 16 clinical signs and symptoms from ED reports, all of which contributed to our ML (HIVE) model (Multimedia Appendix 2). To that end, a 4-step workflow was implemented (Figure 2). First, ED reports were compiled into a JSON file. Next, a base prompt was designed, consisting of an instruction specifying which symptom or sign to extract and an output with a predefined set of answer options. From this base prompt, two 0-shot prompting strategies were derived. The minimal prompt strategy extended the base prompt with a limited set of essential clarifications, identical to the annotation rules provided to ED physicians to ensure equal comparison. The optimized prompt strategy further integrated additional elements aimed at steering performance, including broader context (artificial intelligence [AI] persona and report type), additional instructions (section limitations, and negations or constraints of symptoms), and specific context (domain-specific terminology or abbreviations, and symptom explanations). To quantify the contribution of individual elements, an ablation study was conducted at the start of the study on a development set of 100 ED reports. Each element was sequentially added to the base prompt, and performance was compared against the researcher’s annotations. Only elements that improved performance were included in the optimized prompt. Both prompts used the same annotation rules; any differences between them are due to the prompt design, not to different instructions. All prompts and reports were provided to the LLM in Dutch. The LLM processed each report from the development (n=100) and evaluation sets (n=236), producing structured output in JSON format. The JSON format consisted of a pseudonymized patient identifier, the name of the target symptom, and the predefined answer options of the target symptom (eg, {“uid”: “82547948A6A5E91B93B2BAACAE3F943508FD7EFA,” “abdominal pain location”: [“right lower quadrant,” “diffuse”]}). Third, features extracted from 68 of 236 evaluation reports were combined with structured intake data and vital signs. Finally, these datasets were used for ML model validation. The remaining 268 cases, annotated by the research team, were used for developing the ML model.

### LLM Selection

In December 2024 and January 2025, four popular LLMs available on the Ollama platform were evaluated using 4 representative features varying in complexity and output type. Selected models, Mistral-Nemo 12B Instruct [20], Qwen2.5:14B [21], DeepSeek R1:14B distilled from Qwen2.5 [22], and Gemma2:9B [23], were chosen for their multilingual capabilities, input length, and compact size (<10 GB), allowing deployment on a 12 GB GPU laptop without requiring connection to a computational cluster, which may be impractical in ED settings.

After evaluation, Alibaba’s Qwen2.5:14B Instruct was selected based on performance on the development set (n=100). All LLMs were assessed on the same data, prompts, and settings (eg, temperature=0.1), supporting deterministic output [24]. Ollama was chosen for its capability to facilitate local LLM deployment. The underlying LLM extraction tool for this study is available in a repository (10.6084/m9.figshare.28931030) and on GitHub [25] with a complete walkthrough to adopt the framework and tooling, and incorporates components from a previously published data extraction repository [26].

### Postprocessing of LLM Outputs

LLM outputs were checked against the predefined answer options. To mimic daily practice, where manual postprocessing is infeasible, responses outside these options were removed. Occasionally, the LLM returned descriptive terms accurately copied from the ED report but not listed among the predefined answers. These nonconforming terms were removed, and the feature was labeled as “not reported.” All performance metrics presented reflect this postprocessing step.

### Reader Study–Manual Labeling

A reader study was conducted to compare LLM outputs, under the minimal and optimized prompt strategies, with manual labeling of 2 ED physicians using the same evaluation set (n=236). Each ED report was presented in its original format and independently reviewed by 2 ED physicians, one with 2 years of residency experience and one with 5 years of postqualification experience. Physicians labeled 16 clinical signs and symptoms using an Excel (Microsoft Corp) spreadsheet. Predefined answer options matched those given to the LLM and were provided as drop-down menus: single-choice for binary and multiclass features, and multiple-choice for multilabel features. If a sign or symptom was absent or not reported, the field was left blank.

### Statistical Analysis

LLM- and physician-labeled features were evaluated using specificity, sensitivity, and accuracy. The HIVE model’s performance was assessed via AUROCs. For multiclass and multilabel features, LLM performance metrics were calculated for each positive class and averaged over all positive classes. 95% CIs were estimated through bootstrapping with replacement.

### ML Model Development and Validation

An XGBoost (version 2.1.1) algorithm was selected to estimate the probability of appendicitis, following the approach described in our previous study [18,27]. Referred to as the HIVE model, it incorporates ED intake information, vital signs, medical history, and physical examination features. As explained in this study’s design, only routinely measured vital signs and intake information, combined with medical history and physical examination features that contributed most to our previous HIVE model, were included in this model. Following the same set-up, 336 cases were divided into 268 cases for training or tuning (researcher-labeled), and 68 validation cases relabeled by the LLM, under 2 prompting strategies, and by 2 ED physicians. This allowed comparison against the original reference annotations.

For model tuning, repeated stratified 10-fold cross-validation was used to preserve class distributions. Categorical parameters were encoded using CatBoost (version 2.6.3) into numerical representations derived from training data [28,29]. Hyperparameters were optimized to maximize AUROC using Bayesian optimization with Optuna (version 3.6.1) over 100 trials [30]. The optimized hyperparameters were a learning rate (η) of 0.012, a maximum tree depth of 3, a minimum sum of weights in a child of 4, a subsample ratio of features for each tree of 0.64, and 156 boosting rounds. Class weights were not adjusted, given that the dataset was approximately balanced (167 appendicitis vs 169 other AAP causes). Feature contributions to model predictions were quantified using TreeSHAP (version 0.46.0). To examine whether downstream performance depended on model choice, we trained a random forest on the same 236 researcher-labeled cases and evaluated it on the same 68-case validation set using the LLM-extracted features. Preprocessing matched the HIVE pipeline, with numeric missing values imputed using an Iterative Imputer (max_iter=10) for compatibility with the random forest model. A standard configuration was used (n_estimators=500, max_depth=None, bootstrap=True). The TRIPOD (Transparent Reporting of a Multivariable Prediction Model for Individual Prognosis or Diagnosis) checklist was followed to ensure methodological transparency (Checklist 1), and all code including the LLM extraction tool are available via figshare (10.6084/m9.figshare.28931030).

### Ethical Considerations

This study was conducted according to the Declaration of Helsinki and Guidelines for Good Clinical Practice. The execution of this retrospective observational study of patient records was approved by the local review board of the Jeroen Bosch Hospital (number 2023.11.22.02), and was judged by the Medical Research Ethics Committee Brabant, which waived this study to be subject to the regulations of the Dutch Medical Research Involving Human Subjects Act, including a waiver of informed written consent and a consent for publication (Medical Research Ethics Committee number NW2024-05) [31].

Data were extracted from the EHR using CTcue (IQVIA) and handled in a pseudonymized manner. No directly identifiable patient information was accessed by the researchers. Data extraction and analysis were performed in a secure environment in accordance with institutional and European and national data protection regulations. Participants received no compensation.

## Results

### Overview

Among 336 patients with AAP (median age 41, IQR 22‐62 years, 205/336, 61% were female), 50% (167/336) had appendicitis (Table 1, Table 2). Reports were authored by various physicians over 7 years. Feature prevalence ranged from 6% (n=20) for pollakiuria to 91% (n=303) for pain location (Table 3).

**Table 1.** Categorical patient characteristics (n=336). Structured EHR^a^ data (intake and vital signs).

	Feature	Section	Class	Values, n (%)
1	Sex	Intake	Female	205 (61.0)
2	Transport	Intake		
			Ambulance	32 (9.5)
			Own transport	303 (90.2)
			Other	1 (0.0)
3	Referrer	Intake		
			Primary care physician	219 (65.2)
			Self-referral	98 (29.2)
			Hospital	9 (2.7)
			Ambulance	5 (1.5)
			Other facility	1 (0.0)
			Not reported	4 (1.2)
4	EMV^b^	Vital signs		201 (59.8)
5	Q-SOFA^c^	Vital signs		103 (30.7)

a EHR: electronic health record.

b EMV: Eye Opening, Best Motor Response, Best Verbal Response.

c Q-SOFA: quick sepsis-related organ failure assessment

**Table 2.** Numeric patient characteristics (n=336). Structured EHR^a^ data (intake and vital signs).

	Feature	Section	Median (IQR)	Values, n (%)
1	Age	Intake	41 (22‐62)	336 (100.0)
2	Prior ED^b^ visits with AAP^c^	Intake	1 (1-1)	336 (100.0)
3	Pain rating	Intake	6 (4-8)	291 (86.6)
4	Diastolic arterial pressure (mm Hg)	Vital signs	79 (71‐88)	334 (99.4)
5	Systolic arterial pressure (mm Hg)	Vital signs	130 (118‐144)	326 (97.0)
6	Mean arterial pressure (mm Hg)	Vital signs	96 (88‐105)	316 (94.0)
7	Respiratory rate (rpm)	Vital signs	16 (15‐19)	132 (39.3)
8	Heart rate (bpm)	Vital signs	85 (74‐98)	316 (94.0)
9	SIRS^d^	Vital signs	0 (0‐1)	189 (56.3)
10	Oxygen saturation	Vital signs	98 (97‐100)	334 (99.4)
11	Temperature (°C)	Vital signs	37.1 (36.6‐37.5)	333 (99.4)

a EHR: electronic health record.

b ED: emergency department.

c AAP: acute abdominal pain.

d SIRS: systematic inflammatory response syndrome.

**Table 3.** Prevalence of 16 clinical features in ED^a^ reports (medical history and physical examination; n=336).

	Feature	Section	Classification	Class	Class prevalence, n (%)	Feature prevalence, n (%)
1	Abdominal pain location	Physical examination	Multilabel	Right lower quadrant	222 (67.0)	301 (89.6)
				Left lower quadrant	32 (9.5)	
				Right upper quadrant	28 (8.3)	
				Left upper quadrant	9 (2.7)	
				Epigastric region	18 (5.4)	
				Hypogastric region	11 (3.3)	
				Periumbilical	27 (8.0)	
				Diffuse	21 (6.3)	
2	Nausea	Medical history	Binary		177 (52.7)	177 (52.7)
3	Development of complaints	Medical history	Multilabel	Increase	132 (39.2)	150 (44.6)
				Decrease	40 (11.9)	
4	Onset of pain	Medical history	Multiclass	Acute	56 (16.7)	63 (18.8)
				Gradual	7 (2.1)	
5	McBurney’s sign	Physical examination	Binary		118 (35.1)	118 (35.1)
6	Rebound tenderness	Physical examination	Binary		100 (29.7)	100 (29.7)
7	Anorexia	Medical history	Binary		122 (36.3)	122 (36.3)
8	Pain migration to the right lower quadrant	Medical history	Binary		77 (22.9)	77 (22.9)
9	Fever	Medical history	Binary		70 (20.8)	70 (20.8)
10	Abdominal inspection	Physical examination	Multilabel	Adipose	44 (13.1)	131 (39.0)
				Scars	22 (6.5)	
				Distended abdomen	25 (7.4)	
				No abnormalities	49 (14.6)	
11	Pollakiuria	Medical history	Binary		20 (6.0)	20 (6.0)
12	Stool consistency	Medical history	Multilabel	Diarrhea	39 (11.6)	257 (76.5)
				Loose	62 (18.5)	
				Normal	154 (45.8)	
				Stiff or obstipation	31 (9.2)	
13	Pain location	Medical history	Multilabel	Right lower quadrant	166 (49.4)	303 (91.2)
				Left lower quadrant	64 (19.0)	
				Right upper quadrant	29 (8.6)	
				Left upper quadrant	18 (5.4)	
				Epigastric region	34 (10.1)	
				Hypogastric region	9 (2.7)	
				Periumbilical	71 (21.1)	
				Diffuse	25 (7.4)	
				Flank right	8 (2.4)	
				Flank left	4 (1.2)	
				Flanks both sides	3 (1.0)	
14	Pain manifestation	Medical history	Multilabel	Attack-wise	71 (21.1)	180 (53.6)
				Continuous	138 (41.1)	
15	Nature of pain	Medical history	Multilabel	Aching	46 (13.7)	122 (36.3)
				Cramping	34 (10.1)	
				Stabbing	66 (19.6)	
				Burning	2 (0.5)	
16	Palpation tenderness (supple)	Physical examination	Binary		236 (70.2)	236 (70.2)

a ED: emergency department.

### Ablation Study

An ablation study on the development set (n=100) quantified the contribution of individual prompt elements (Tables4^6^). Sequentially adding each element to the base prompt showed the largest performance gains for negation or constraint, domain-specific context, and explanation, while section limitation, AI persona, and report type produced smaller and less consistent improvements across features (Tables5  6). These findings are incorporated into the composition of the optimized prompts (Table 4). Some clarifying elements, particularly negation or constraint elements, were also integrated into the minimal prompt to ensure an equal comparison with manual annotations by ED physicians. Certain elements were not applicable to specific features (eg, when no negation or explanation was possible) and are therefore left blank in Tables5  6. Table 7 lists the minimal prompts used to extract each of the 16 features; these incorporate the same annotation rules that were provided to ED physicians. Table 8 presents the corresponding optimized prompts, which integrate the prompt elements identified in the ablation study as improving extraction performance. Multimedia Appendix 3 presents all prompt elements evaluated for each feature in the ablation study.

**Table 4.** Ablation study (development set, n=100). Performance of base, minimal, and optimized prompting strategies.

	Prompting strategies			Base (instruction + output)			Minimal			Optimized		
	Feature	Section	Classes	Specificity ±CI	Sensitivity ±CI	Accuracy ± CI	Specificity ± CI	Sensitivity ± CI	Accuracy ± CI	Specificity ± CI	Sensitivity ± CI	Accuracy ± CI
1	Abdominal pain location	Physical examination	8	0.93 ± 0.02	0.78 ± 0.07	0.91 ± 0.02	0.95 ± 0.02	0.78 ± 0.07	0.93 ± 0.02	0.96 ± 0.01	0.89 ± 0.06	0.95 ± 0.01
2	Nausea	Medical history	1	0.85 ± 0.11	0.67 ± 0.12	0.76 ± 0.08	0.92 ± 0.07	0.67 ± 0.12	0.80 ± 0.07	0.96 ± 0.05	0.94 ± 0.07	0.95 ± 0.04
3	Development of complaints	Medical history	2	0.89 ± 0.05	0.86 ± 0.09	0.88 ± 0.05	0.89 ± 0.05	0.86 ± 0.09	0.88 ± 0.05	0.91 ± 0.04	0.86 ± 0.09	0.90 ± 0.04
4	Onset of pain	Medical history	2	0.64 ± 0.07	0.94 ± 0.08	0.67 ± 0.07	0.64 ± 0.07	0.94 ± 0.08	0.67 ± 0.07	0.67 ± 0.07	0.89 ± 0.14	0.69 ± 0.06
5	McBurney’s sign	Physical examination	1	0.80 ± 0.10	0.97 ± 0.04	0.86 ± 0.07	0.91 ± 0.07	0.94 ± 0.07	0.92 ± 0.05	0.94 ± 0.05	0.97 ± 0.04	0.95 ± 0.04
6	Rebound tenderness	Physical examination	1	0.93 ± 0.06	1.00 ± 0.00	0.95 ± 0.04	0.96 ± 0.05	1.00 ± 0.00	0.97 ± 0.03	—^a^	—^a^	—^a^
7	Anorexia	Medical history	1	0.98 ± 0.02	0.95 ± 0.06	0.97 ± 0.03	0.98 ± 0.02	0.95 ± 0.06	0.97 ± 0.03	—^a^	—^a^	—^a^
8	Pain migration to right lower quadrant	Medical history	1	0.90 ± 0.07	0.95 ± 0.07	0.91 ± 0.05	0.90 ± 0.07	0.95 ± 0.07	0.91 ± 0.05	—^a^	—^a^	—^a^
9	Fever	Medical history	1	0.82 ± 0.08	0.95 ± 0.07	0.85 ± 0.07	0.83 ± 0.08	0.95 ± 0.07	0.86 ± 0.07	—^a^	—^a^	—^a^
10	Abdominal inspection	Physical examination	4	1.00 ± 0.00	0.88 ± 0.09	0.98 ± 0.01	1.00 ± 0.00	0.88 ± 0.09	0.98 ± 0.01	0.99 ± 0.01	0.98 ± 0.03	0.99 ± 0.01
11	Pollakiuria	Medical history	1	0.98 ± 0.03	0.50 ± 0.33	0.95 ± 0.03	0.98 ± 0.03	0.50 ± 0.33	0.95 ± 0.03	0.98 ± 0.03	1.00 ± 0.00	0.98 ± 0.03
12	Stool consistency	Medical history	4	0.97 ± 0.02	0.67 ± 0.10	0.91 ± 0.03	0.97 ± 0.02	0.67 ± 0.10	0.91 ± 0.03	0.96 ± 0.02	0.87 ± 0.07	0.94 ± 0.02
13	Pain location	Medical history	11	0.94 ± 0.01	0.70 ± 0.08	0.91 ± 0.02	0.95 ± 0.01	0.79 ± 0.07	0.93 ± 0.01	0.96 ± 0.01	0.83 ± 0.07	0.94 ± 0.01
14	Pain manifestation	Medical history	2	0.77 ± 0.07	0.78 ± 0.10	0.77 ± 0.06	0.77 ± 0.07	0.78 ± 0.10	0.77 ± 0.06	0.94 ± 0.04	0.78 ± 0.10	0.90 ± 0.04
15	Nature of pain	Medical history	4	0.96 ± 0.02	0.95 ± 0.06	0.96 ± 0.02	0.96 ± 0.02	0.95 ± 0.06	0.96 ± 0.02	0.98 ± 0.02	0.95 ± 0.06	0.97 ± 0.02
16	Palpation tenderness (supple)	Physical examination	1	1.00 ± 0.00	1.00 ± 0.00	1.00 ± 0.00	1.00 ± 0.00	1.00 ± 0.00	1.00 ± 0.00	—^a^	—^a^	—^a^

a No prompt element improved performance compared with the minimal prompt; the minimal prompt was retained.

**Table 5.** Ablation study (development set, n=100). Incremental effect of individual prompt elements on extraction performance relative to the base prompt.

	Prompt element^a^	AI^b^ persona			Report type			Section limitation		
	Feature	Specificity ± CI	Sensitivity ± CI	Accuracy ± CI	Specificity ± CI	Sensitivity ± CI	Accuracy ± CI	Specificity ± CI	Sensitivity ± CI	Accuracy ± CI
1	Abdominal pain location	0.93 ± 0.02^c^	0.79 ± 0.07^c^	0.91 ± 0.02^c^	0.94 ± 0.02	0.75 ± 0.08	0.91 ± 0.02	0.95 ± 0.02	0.75 ± 0.08	0.92 ± 0.02
2	Nausea	0.88 ± 0.09^c^	0.65 ± 0.12^c^	0.77 ± 0.08^c^	0.90 ± 0.09^c^	0.65 ± 0.12^c^	0.78 ± 0.08^c^	0.83 ± 0.11	0.69 ± 0.12	0.76 ± 0.08
3	Development of complaints	0.84 ± 0.06	0.86 ± 0.09	0.84 ± 0.05	0.83 ± 0.06	0.86 ± 0.09	0.83 ± 0.05	0.71 ± 0.07	0.82 ± 0.11	0.74 ± 0.06
4	Onset of pain	0.62 ± 0.07	0.94 ± 0.08	0.65 ± 0.07	0.62 ± 0.07	0.94 ± 0.08	0.65 ± 0.07	0.57 ± 0.07	1.00 ± 0.00	0.60 ± 0.07
5	McBurney’s sign	0.80 ± 0.10	0.97 ± 0.04	0.86 ± 0.07	0.80 ± 0.10	0.97 ± 0.04	0.86 ± 0.07	0.75 ± 0.11	0.97 ± 0.04	0.83 ± 0.07
6	Rebound tenderness	0.93 ± 0.06	1.00 ± 0.00	0.95 ± 0.04	0.91 ± 0.06	1.00 ± 0.00	0.94 ± 0.04	0.91 ± 0.06	1.00 ± 0.00	0.94 ± 0.04
7	Anorexia	0.98 ± 0.02	0.95 ± 0.06	0.97 ± 0.03	0.98 ± 0.02	0.92 ± 0.08	0.96 ± 0.03	0.98 ± 0.02	0.95 ± 0.06	0.97 ± 0.03
8	Pain migration to the right lower quadrant	0.68 ± 0.10	0.95 ± 0.07	0.74 ± 0.08	0.73 ± 0.10	0.95 ± 0.07	0.78 ± 0.08	0.87 ± 0.07	1.00 ± 0.00	0.90 ± 0.05
9	Fever	0.82 ± 0.08	0.95 ± 0.07	0.85 ± 0.07	0.82 ± 0.08	0.95 ± 0.07	0.85 ± 0.07	0.82 ± 0.08	0.95 ± 0.07	0.85 ± 0.07
10	Abdominal inspection	0.99 ± 0.01	0.91 ± 0.08	0.98 ± 0.01	0.99 ± 0.01	0.91 ± 0.08	0.98 ± 0.01	0.99 ± 0.01	0.88 ± 0.09	0.98 ± 0.01
11	Pollakiuria	0.98 ± 0.03	0.50 ± 0.33	0.95 ± 0.03	0.98 ± 0.03	0.50 ± 0.33	0.95 ± 0.03	0.98 ± 0.03	0.50 ± 0.33	0.95 ± 0.03
12	Stool consistency	0.97 ± 0.02	0.66 ± 0.10	0.90 ± 0.03	0.98 ± 0.01	0.67 ± 0.10	0.91 ± 0.03	0.96 ± 0.02	0.74 ± 0.09	0.91 ± 0.03
13	Pain location	0.93 ± 0.02	0.69 ± 0.08	0.91 ± 0.02	0.94 ± 0.02	0.73 ± 0.08	0.91 ± 0.02	0.94 ± 0.01^c^	0.76 ± 0.07^c^	0.92 ± 0.02^c^
14	Pain manifestation	0.74 ± 0.07	0.81 ± 0.10	0.77 ± 0.06	0.77 ± 0.07	0.78 ± 0.10	0.77 ± 0.06	0.63 ± 0.08	0.76 ± 0.11	0.67 ± 0.06
15	Nature of pain	0.98 ± 0.02^c^	0.95 ± 0.06^c^	0.97 ± 0.02^c^	0.91 ± 0.03	0.95 ± 0.06	0.92 ± 0.03	0.88 ± 0.03	0.98 ± 0.03	0.89 ± 0.03
16	Palpation tenderness (supple)	—^d^	—^d^	—^d^	—^d^	—^d^	—^d^	—^d^	—^d^	—^d^

a Each prompt element shows the isolated incremental effect compared to the base prompt.

b AI: artificial intelligence.

c Element retained in the optimized prompt configuration.

d Prompt element was not applicable and evaluated for that specific variable.

**Table 6.** Ablation study (development set, n=100). Incremental effect of individual prompt elements on extraction performance relative to the base prompt.

	Prompt element^a^	Negation or constraint			Domain-specific context			Explanation		
	Feature	Specificity ± CI	Sensitivity ± CI	Accuracy ± CI	Specificity ± CI	Sensitivity ± CI	Accuracy ± CI	Specificity ± CI	Sensitivity ± CI	Accuracy ± CI
1	Abdominal pain location	0.95 ± 0.02^b^ ^c^	0.78 ± 0.07^b^ ^c^	0.93 ± 0.02^b^ ^c^	0.94 ± 0.02^c^	0.87 ± 0.06^c^	0.93 ± 0.02^c^	0.94 ± 0.02^c^	0.78 ± 0.07^c^	0.92 ± 0.02^c^
2	Nausea	0.92 ± 0.07^b^ ^c^	0.67 ± 0.12^b^ ^c^	0.80 ± 0.07^b^ ^c^	0.87 ± 0.09^c^	0.81 ± 0.10^c^	0.84 ± 0.07^c^	0.90 ± 0.09^c^	0.85 ± 0.09^c^	0.88 ± 0.06^c^
3	Development of complaints	0.91 ± 0.04^b^ ^c^	0.86 ± 0.09^b^ ^c^	0.90 ± 0.04^b^ ^c^	0.78 ± 0.07	0.90 ± 0.09	0.81 ± 0.05	—^d^	—^d^	—^d^
4	Onset of pain	0.67 ± 0.07^c^	0.89 ± 0.14^c^	0.69 ± 0.06^c^	0.62 ± 0.07	1.00 ± 0.00	0.65 ± 0.07	0.59 ± 0.07	0.94 ± 0.08	0.62 ± 0.06
5	McBurney’s sign	0.91 ± 0.07^b^ ^c^	0.94 ± 0.07^b^ ^c^	0.92 ± 0.05^b^ ^c^	0.91 ± 0.07^c^	0.94 ± 0.07^c^	0.92 ± 0.05^c^	0.73 ± 0.11	0.94 ± 0.07	0.81 ± 0.08
6	Rebound tenderness	0.96 ± 0.05^b^ ^c^	1.00 ± 0.00^b^ ^c^	0.97 ± 0.03^b^ ^c^	—^d^	—^d^	—^d^	0.93 ± 0.06	1.00 ± 0.00	0.95 ± 0.04
7	Anorexia	—^d^	—^d^	—^d^	0.95 ± 0.06	0.97 ± 0.04	0.96 ± 0.03	0.95 ± 0.06	0.97 ± 0.04	0.96 ± 0.03
8	Pain migration to the right lower quadrant	0.72 ± 0.10	0.95 ± 0.07	0.77 ± 0.08	—^d^	—^d^	—^d^	0.87 ± 0.07	1.00 ± 0.00	0.90 ± 0.05
9	Fever	0.78 ± 0.10	0.91 ± 0.11	0.81 ± 0.08	0.83 ± 0.08^b^ ^c^	0.95 ± 0.07^b^ ^c^	0.86 ± 0.07^b^ ^c^	0.74 ± 0.10	0.95 ± 0.07	0.79 ± 0.08
10	Abdominal inspection	1.00 ± 0.00	0.88 ± 0.09	0.98 ± 0.01	0.99 ± 0.01^c^	0.98 ± 0.03^c^	0.99 ± 0.01^c^	0.99 ± 0.01	0.88 ± 0.09	0.98 ± 0.01
11	Pollakiuria	—^d^	—^d^	—^d^	0.98 ± 0.03^c^	1.00 ± 0.00^c^	0.98 ± 0.03^c^^d^	0.98 ± 0.03	0.50 ± 0.33	0.95 ± 0.03
12	Stool consistency	—^d^	—^d^	—^d^	0.97 ± 0.02^c^	0.81 ± 0.08^c^	0.94 ± 0.02^c^	0.97 ± 0.02^c^	0.73 ± 0.09^c^	0.92 ± 0.03^c^
13	Pain location	0.95 ± 0.01^b^ ^c^	0.79 ± 0.07^b^ ^c^	0.93 ± 0.01^b^ ^c^	0.94 ± 0.01^c^	0.78 ± 0.07^c^	0.93 ± 0.02^c^	—^d^	—^d^	—^d^
14	Pain manifestation	0.92 ± 0.05^c^	0.75 ± 0.11^c^	0.87 ± 0.05^c^	0.72 ± 0.07	0.83 ± 0.09	0.75 ± 0.06	0.79 ± 0.07^c^	0.80 ± 0.10^c^	0.80 ± 0.05^c^
15	Nature of pain	0.96 ± 0.02	0.95 ± 0.06	0.96 ± 0.02	—^d^	—^d^	—^d^	—^d^	—^d^	—^d^
16	Palpation tenderness (supple)	—^d^	—^d^	—^d^	—^d^	—^d^	—^d^	—^d^	—^d^	—^d^

a Each prompt element shows the isolated incremental effect compared to the base prompt.

b Element retained in the minimal prompt configuration. (Additional instruction shared with physician)

c Element retained in the optimized prompt configuration.

d Prompt element was not applicable and evaluated for that specific variable.

**Table 7.** Minimal 0-shot prompts as presented to the LLM.^a^

	Feature	Prompt^b^
1	Abdominal pain location	Report where abdominal pain is present in this patient. If multiple locations of abdominal pain are mentioned, report the most painful location (PM^c^).^d^ Limit your answer to the following options: “right lower quadrant,” “right upper quadrant,” “left lower quadrant,” “left upper quadrant,” “entire upper abdomen,” “entire lower abdomen,” “entire left abdomen,” “entire right abdomen,” “periumbilical,” “epigastric,” “hypogastric,” “diffuse,” or “absent.” Report “absent” if abdominal pain is not mentioned or explicitly absent.
2	Nausea	Report whether this patient has nausea based on the last status in the report. Ignore vomiting. Vomiting is not the same as nausea.^d^ Limit your answer to the following options: “yes” or “no.” Report “no” if nausea is not mentioned or explicitly absent.
3	Development of complaints	Report whether there is an increase or decrease in the development of complaints after the onset in this patient. A decrease in the development of complaints occurring after the administration of pain medication must be ignored and should not be reported as a decrease.^d^ Limit your answer to the following options: “increase,” “decrease,” or “not reported.” If none of the mentioned options are explicitly stated in the text, the answer should be “not reported.”
4	Onset of pain	Report whether there is a description of how the onset of pain started in this patient. Limit your answer to the following options: “acute,” “gradual,” or “not reported.” If none of the mentioned options are explicitly stated in the text, the answer should be “not reported.”
5	McBurney’s sign	Report whether this patient has abdominal pain at McBurney’s point. If McBurney’s sign is dubious, report “no.”^d^ Limit your answer to the options: “yes” or “no.” Report “no” if abdominal pain at McBurney's point is not mentioned or explicitly absent.
6	Rebound tenderness	Report whether this patient has rebound tenderness. Ignore contralateral rebound tenderness.^d^ Limit your answer to the options: “yes” or “no.” Report “no” if rebound tenderness is not mentioned or explicitly absent.
7	Anorexia	Report whether this patient has anorexia. Limit your answer to the options: “yes” or “no.” Report "no” if anorexia is not mentioned or explicitly absent.
8	Pain migration to the right lower quadrant	Report whether the abdominal pain has migrated to the right lower quadrant in the patient. Limit your answer to the options: “yes” or “no.” Report “no” if migration to the right lower quadrant is not mentioned or explicitly absent
9	Fever	Report whether this patient has a fever. Limit your answer to the options: “yes” or “no.” Note that the term may not be mentioned exactly as written. Yes=recorded elevated temperature, feeling of fever, measured fever, feverish, temperature or “T” of 38°C or higher. No=temperature or “T” of 37.9°C or lower, no fever, not feverish, no elevated temperature.^d^ Report “no” if fever is not mentioned or explicitly absent.
10	Abdominal inspection	Report whether any abnormalities are noted in the inspection of the abdomen for this patient. Limit your answer to the following options: “adipose,” “distended abdomen,” “scars,” “no abnormalities,” or “not reported.” If none of the mentioned options are explicitly stated in the text, answer “not reported.”
11	Pollakiuria	Report whether this patient has pollakiuria. Limit your answer to the options: “yes” or “no.” Report “no” if pollakiuria is not mentioned or explicitly absent.
12	Stool consistency	Report whether stool consistency is described for this patient. Limit your answer to the following options: “diarrhea,” “loose,” “normal,” “stiff/obstipation,” or “not reported.” If none of the mentioned options are explicitly stated in the text, answer “not reported.”
13	Pain location	Report where the pain initially started in this patient. Ignore pain resulting from displacement, migration, or radiation. Ignore abdominal tenderness. Ignore findings from the physical examination.^d^ Limit your answer to the following options: “right lower quadrant,” “right upper quadrant,” “left lower quadrant,” “left upper quadrant,” “entire upper abdomen,” “entire lower abdomen,” “entire left abdomen,” “entire right abdomen,” “around the navel,” “epigastric,” “hypogastric,” “diffuse,” “bilateral flanks,” “right flank” “left flank,” and “absent.” Report “absent” if the location where the pain initially started is not mentioned.
14	Pain manifestation	Report whether this patient has a specific abdominal pain pattern. Limit your answer to the following options: “attack wise,” “continuous,” or “not reported.” If none of the mentioned options are explicitly stated in the text, answer “not reported.”
15	Nature of pain	Report whether the nature of pain is described for this patient. Limit your answer to the following options: “stabbing,” “aching,” “cramping,” “burning,” or “not reported.” If none of the mentioned options are explicitly stated in the text, answer “not reported.”
16	Palpation tenderness	Report whether this patient has a supple abdomen upon palpation. Limit your answer to the options: “yes” or “no.” Report “no” if a supple abdomen upon palpation is not mentioned or explicitly absent.

a LLM: large language model.

b All prompts and emergency department reports were provided to the large language model in Dutch and have been translated into English for readability.

c PM: punctum maximum.

d Annotation rules shared with or instructed to emergency department physicians for manual labeling and included in the minimal prompt.

**Table 8.** Optimized 0-shot prompts as presented to the LLM.^a^

	Feature	Prompt^b^
1	Abdominal pain location	You are a medical information extraction specialist.^c^ Report where abdominal pain is present in this patient. If multiple locations of abdominal pain are mentioned, report the most painful location (punctum maximum/PM).^d^ Limit your answer to the following options: “right lower quadrant,” “right upper quadrant,” “left lower quadrant,” “left upper quadrant,” “entire upper abdomen,” “entire lower abdomen,” “entire left abdomen,” “entire right abdomen,” “periumbilical,” “epigastric,” “hypogastric,” “diffuse,” or “absent.” Note that these terms may not be mentioned exactly as listed. Right lower quadrant=RLQ, RLA, McBurney, right iliac fossa. Right upper quadrant=RUQ, Murphy. Left lower quadrant=LLQ, LLA, left iliac fossa. Left upper quadrant=LUQ. Entire right abdomen=right hemiabdomen. Entire left abdomen=left hemiabdomen. Periumbilical=around the navel, paraumbilical, supraumbilical. Epigastric=stomach region. Hypogastric=uterus, suprapubic, above pubis. Diffuse=entire abdomen. Absent=no abdominal pain. During the physical examination, the physician presses on all these locations and describes where pain is felt.^e^ Report “absent” if pain is not mentioned or explicitly absent.
2	Nausea	You are a medical information extraction specialist.^c^ Your task is to report from the emergency department report whether^c^ this patient has nausea based on the last status in the report. Ignore vomiting. Vomiting is not the same as nausea.^d^ Limit your answer to the following options: “yes” or “no.” Note that these terms may not be mentioned exactly as listed. Yes=N+, N+. No=N−, N−. “N” stands for nausea. “N” often appears in combination with “V,” for example, “N+ V−.” “+” means nausea is present. “−” means nausea is absent.^e^ Report “no” if nausea is not mentioned or explicitly absent.
3	Development of complaints	Report whether there is an increase or decrease in the development of complaints after the onset in this patient. A decrease in the development of complaints occurring after the administration of pain medication must be ignored and should not be reported as a decrease.^d^ Ignore migration and radiation of abdominal pain. These do not count as an increase in the progression of abdominal pain complaints.^f^ Limit your answer to the following options: “increase,” “decrease,” or “not reported.” If none of the mentioned options are explicitly stated in the text, the answer should be “not reported.”
4	Onset of pain	Report whether there is a description of how the onset of pain started in this patient. Ignore the timing of onset and focus only on how the complaints began.^f^ Limit your answer to the following options: “acute,” “gradual,” or “not reported.” If none of the mentioned options are explicitly stated in the text, the answer should be “not reported.”
5	McBurney’s sign	Report whether this patient has abdominal pain at McBurney's point. If McBurney's sign is dubious, report “no.”^d^ Limit your answer to the options: “yes” or “no.” Note that the term may not be mentioned exactly as listed. Yes=McBurney +, PM McBurney, punctum maximum McBurney. No=McBurney −, no pain at McBurney's point, not at McBurney.^e^ Report “no” if abdominal pain at McBurney's point is not mentioned or explicitly absent.
6	Rebound tenderness	—^g^
7	Anorexia	—^g^
8	Pain migration to right lower quadrant	—^g^
9	Fever	—^g^
10	Abdominal inspection	Report whether any abnormalities are noted in the inspection of the abdomen for this patient. Limit your answer to the following options: “adipose,” “distended abdomen,” “scars,” “no abnormalities,” or “not reported.” Note that the terms may not be mentioned exactly as listed: adipose=obese, adiposity. Distended abdomen=bloated, distended, tense. Scar=Pfannenstiel.^e^ If none of the mentioned options are explicitly stated in the text, answer “not reported.”
11	Pollakiuria	Report whether this patient has pollakiuria. Limit your answer to the options: “yes” or “no.” Note that the term may not be mentioned exactly as listed: Yes=urinating in small amounts, frequent urination, urinating more often than normal, pollakiuria +. No=pollakiuria −.^e^ Report “no” if pollakiuria is not mentioned or explicitly absent.
12	Stool consistency	Report whether the stool consistency is described for this patient. Limit your answer to the following options: “diarrhea,” “loose,” “normal,” “stiff/obstipation,” or “not reported.” Note that the terms may not be mentioned exactly as listed: diarrhea=watery, liquid stool, extremely loose, explosive. Normal=na (no abnormalities), no notable findings, no deviations, no diarrhea or obstipation. Constipation=difficult passage. Stool may also be referred to as defecation or def.^e^ If none of the mentioned options are explicitly stated in the text, answer “not reported.”
13	Pain location	Report where the pain initially started in this patient. Limit yourself to the medical history section.^c^ Ignore pain resulting from displacement, migration, or radiation. Ignore abdominal tenderness. Ignore findings from the physical examination.^d^ Limit your answer to the following options: “right lower quadrant,” “right upper quadrant,” “left lower quadrant,” “left upper quadrant,” “entire upper abdomen,” “entire lower abdomen,” “entire left abdomen,” “entire right abdomen,” “around the navel,” “epigastric,” “hypogastric,” “diffuse,” “bilateral flanks,” “right flank,” “left flank,” “absent.” Note that the terms may not be mentioned exactly as listed: right lower quadrant=RLQ, RLA. Right upper quadrant=RUQ. Left lower quadrant=LLQ, LLA. Left upper quadrant=LUQ. Around the navel=periumbilical, para-umbilical. Entire right abdomen=right hemiabdomen. Entire left abdomen=left hemiabdomen. Entire lower abdomen=lower part of the abdomen. Hypogastric=uterus, suprapubic, above pubis. Epigastric=stomach region. Diffuse=entire abdomen.^e^ Report “absent” if the location where the pain initially started is not mentioned.
14	Pain manifestation	Report whether this patient has a specific abdominal pain pattern. Ignore descriptions of acute, sudden, or stabbing abdominal pain, as these are not specific pain patterns.^f^ Limit your answer to the following options: “attack wise,” “continuous,” or “not reported.” Note that the terms may not be mentioned exactly as written: attack wise=worsening in waves, peak periods, fluctuating, intermittent, variable in intensity, flare-ups, colicky pain, on and off.^e^ If none of the mentioned options are explicitly stated in the text, answer “not reported.”
15	Nature of pain	You are a medical information extraction specialist.^c^ Report whether the nature of pain is described for this patient. Limit your answer to the following options: “stabbing,” “aching,” “cramping,” “burning,” or “not reported.” If none of the mentioned options are explicitly stated in the text, answer “not reported.”
16	Palpation Tenderness	—^g^

a LLM: large language model.

b All prompts and emergency department reports were provided to the large language model in Dutch and have been translated to English for readability.

c Broader context: (1) artificial intelligence persona or (2) report type. These are prompt elements included in the optimized prompt.

d Annotation rules shared with or instructed to emergency department physicians for manual labeling and included in the minimal prompt.

e Specific context: (5) domain-appropriate terminology or abbreviations, or (6) explanation of the symptom.

f Instruction: (3) section limitation or (4) negations or constraints.

g No prompt element improved performance compared with the minimal prompt; the minimal prompt was retained.

### Minimal vs Optimized Prompt Strategies

Table 9 presents the feature-level performance of the LLM under both prompt strategies, evaluated against a reference standard based on lead researcher annotations, shown alongside manual labeling by ED physicians on the evaluation set (n=236). Using the minimal prompt strategy, the LLM achieved an average specificity of 0.929 (95% CI ±0.020), sensitivity of 0.810 (95% CI ±0.058), and accuracy of 0.910 (95% CI ±0.018). Lower feature prevalence in the ED reports did not consistently result in lower LLM accuracy (Table 3). When applying the optimized prompt strategy, performance improved across all metrics; specificity increased to 0.943 (95% CI ±0.017), sensitivity to 0.859 (95% CI ±0.055), and accuracy to 0.929 (95% CI ±0.016). The 5 largest gains in accuracy were observed for nausea, pain manifestation, onset of pain, pain location, and abdominal pain location. No gains appeared for rebound tenderness, anorexia, pain migration to the right lower quadrant, fever, and palpation tenderness. Optimized prompts were particularly beneficial for features involving domain-specific terminology, abbreviations, or overlapping definitions, such as nausea and vomiting.

**Table 9.** Extraction performance of the LLM^a^, under both prompting strategies, vs ED^b^ physicians on the evaluation set (n=236). Each row represents a clinical feature with the number of positive answer options (classes).

		Minimal 0-shot			Optimized 0-shot			ED physician 1			ED physician 2			Comparisons^c^		
Feature	Classes^d^	Spec.^e^ ± CI	Sens.^f^ ± CI	Acc.^g^ ± CI	Spec. ± CI	Sens. ± CI	Acc. ± CI	Spec. ± CI	Sens. ± CI	Acc. ± CI	Spec. ± CI	Sens. ± CI	Acc. ± CI	Δ Acc.^h^	Δ Acc.^i^	Δ Acc.^j^
Abdominal pain location	8	0.96 ± 0.01	0.76 ± 0.05	0.93 ± 0.01	0.96 ± 0.01	0.87 ± 0.04	0.95 ± 0.01	0.99 ± 0.01	0.94 ± 0.03	0.98 ± 0.01	0.96 ± 0.01	0.78 ± 0.05	0.93 ± 0.01	0.02	−0.04	0.01
Nausea	1	0.93 ± 0.05	0.74 ± 0.08	0.82 ± 0.05	0.97 ± 0.03	0.91 ± 0.05	0.94 ± 0.03	0.82 ± 0.07	0.95 ± 0.04	0.89 ± 0.04	1.00 ± 0.00	0.95 ± 0.04	0.98 ± 0.02	0.11	0.05	−0.04
Development of complaints	2	0.88 ± 0.03	0.84 ± 0.06	0.87 ± 0.03	0.91 ± 0.03	0.81 ± 0.07	0.88 ± 0.03	0.99 ± 0.01	0.76 ± 0.07	0.93 ± 0.02	0.98 ± 0.02	0.74 ± 0.08	0.92 ± 0.02	0.01	−0.05	−0.03
Onset of pain	2	0.62 ± 0.08	0.91 ± 0.04	0.65 ± 0.04	0.65 ± 0.04	0.85 ± 0.10	0.67 ± 0.04	0.99 ± 0.01	0.69 ± 0.13	0.96 ± 0.02	0.99 ± 0.01	0.84 ± 0.10	0.98 ± 0.01	0.03	−0.29	−0.31
McBurney’s sign	1	0.97 ± 0.03	1.00 ± 0.00	0.98 ± 0.02	0.99 ± 0.02	0.99 ± 0.02	0.99 ± 0.02	0.99 ± 0.02	0.98 ± 0.03	0.98 ± 0.02	0.99 ± 0.01	0.98 ± 0.03	0.99 ± 0.02	0.01	0.00	0.00
Rebound tenderness	1	0.99 ± 0.01	0.97 ± 0.03	0.97 ± 0.02	0.99 ± 0.01^k^	0.97 ± 0.03^k^	0.97 ± 0.02^k^	1.00 ± 0.00	0.91 ± 0.06	0.98 ± 0.02	0.98 ± 0.02	0.84 ± 0.09	0.94 ± 0.03	0.00	0.00	0.03
Anorexia	1	0.97 ± 0.03	0.83 ± 0.08	0.92 ± 0.04	0.97 ± 0.0^k^	0.83 ± 0.0^k^	0.92 ± 0.04^k^	0.98 ± 0.02	0.81 ± 0.08	0.92 ± 0.03	0.99 ± 0.01	0.59 ± 0.01	0.85 ± 0.04	0.00	0.00	0.07
Pain migration to right lower quadrant	1	0.88 ± 0.05	0.89 ± 0.08	0.88 ± 0.04	0.88 ± 0.05^k^	0.89 ± 0.08^k^	0.88 ± 0.04^k^	0.97 ± 0.03	0.86 ± 0.09	0.95 ± 0.03	0.97 ± 0.03	0.89 ± 0.08	0.95 ± 0.03	0.00	−0.06	−0.07
Fever	1	0.92 ± 0.04	0.77 ± 0.11	0.89 ± 0.04	0.92 ± 0.04^k^	0.77 ± 0.11^k^	0.89 ± 0.04^k^	0.94 ± 0.03	0.92 ± 0.07	0.93 ± 0.03	0.92 ± 0.04	0.98 ± 0.03	0.93 ± 0.03	0.00	−0.05	−0.05
Abdominal inspection	4	0.98 ± 0.01	0.93 ± 0.05	0.98 ± 0.01	0.98 ± 0.01	0.95 ± 0.05	0.98 ± 0.01	0.99 ± 0.01	0.91 ± 0.06	0.98 ± 0.01	1.00 ± 0.00	0.89 ± 0.06	0.99 ± 0.01	0.00	−0.00	−0.01
Pollakiuria	1	1.00 ± 0.00	0.57 ± 0.25	0.98 ± 0.02	1.00 ± 0.00	0.86 ± 0.18	0.99 ± 0.01	1.00 ± 0.00	0.46 ± 0.23	0.97 ± 0.01	1.00 ± 0.01	0.69 ± 0.23	0.98 ± 0.02	0.02	0.02	0.01
Stool consistency	4	0.98 ± 0.01	0.70 ± 0.06	0.92 ± 0.02	0.96 ± 0.02	0.83 ± 0.05	0.93 ± 0.02	0.96 ± 0.02	0.75 ± 0.06	0.91 ± 0.02	0.99 ± 0.01	0.73 ± 0.02	0.94 ± 0.01	0.01	0.02	−0.01
Pain location	11	0.95 ± 0.01	0.75 ± 0.05	0.93 ± 0.01	0.97 ± 0.01	0.80 ± 0.05	0.95 ± 0.01	0.98 ± 0.01	0.85 ± 0.04	0.96 ± 0.01	0.99 ± 0.01	0.71 ± 0.05	0.95 ± 0.01	0.02	−0.02	−0.01
Pain manifestation	2	0.75 ± 0.05	0.85 ± 0.06	0.78 ± 0.04	0.93 ± 0.03	0.79 ± 0.06	0.88 ± 0.03	0.98 ± 0.01	0.87 ± 0.05	0.95 ± 0.02	0.94 ± 0.02	0.91 ± 0.04	0.93 ± 0.02	0.11	−0.06	−0.05
Nature of pain	4	0.95 ± 0.02	0.92 ± 0.05	0.95 ± 0.01	0.95 ± 0.02	0.92 ± 0.05	0.94 ± 0.02	1.00 ± 0.00	0.91 ± 0.05	0.99 ± 0.01	1.00 ± 0.00	0.82 ± 0.05	0.98 ± 0.05	0.00	−0.05	−0.04
Palpation tenderness supple	1	0.96 ± 0.06	0.96 ± 0.03	0.96 ± 0.02	0.96 ± 0.06^k^	0.96 ± 0.03^k^	0.96 ± 0.02^k^	0.93 ± 0.07	0.98 ± 0.02	0.97 ± 0.02	0.94 ± 0.00	0.97 ± 0.07	0.96 ± 0.01	0.00	0.00	0.00
Weighted average^l^	45	0.93 ± 0.02	0.81 ± 0.06	0.91 ± 0.02	0.94 ± 0.02	0.86 ± 0.05	0.93 ± 0.02	0.98 ± 0.01	0.86 ± 0.05	0.96 ± 0.01	0.98 ± 0.01	0.79 ± 0.06	0.95 ± 0.02			

a LLM: large language model.

b ED: emergency department.

c Δ Accuracy columns show pairwise accuracy differences. Δ values indicate improved accuracy of optimized prompts compared with minimal prompts or those of physicians.

d Each class is evaluated as a separate binary classification (present vs absent), meaning the number of binary decisions equals twice the number of classes.

e Spec.: specificity.

f Sens.: sensitivity.

g Acc.: accuracy.

h Accuracy difference (Δ) between minimal vs optimized prompts.

i Accuracy difference (Δ) between emergency department physician 1 vs optimized prompts.

j Accuracy difference (Δ) between emergency department physician 2 vs optimized prompts.

k Optimized prompts were retained only when they improved performance on the development set; otherwise, the minimal prompt was used).

l Weighted averages account for the number of classes per feature. CIs (95% CI) were estimated using bootstrapping.

### LLM vs Physician Labeling

Both physicians achieved higher weighted average specificity and accuracy on the evaluation set than the LLM under either prompting strategy. Physician 1 reached a specificity of 0.978 (95% CI ±0.011), sensitivity of 0.859 (95% CI ±0.015), and accuracy of 0.961 (95% CI ±0.012), while physician 2 achieved a specificity of 0.980 (95% CI ±0.008), sensitivity of 0.793 (95% CI ±0.056), and accuracy of 0.951 (95% CI ±0.015; Table 2).

At the feature level, the LLM using the optimized prompt matched or exceeded the accuracy of physician 1 for 8 of 16 features, and for physician 2 for 9 of 16. Notably, both physicians outperformed the LLM on the onset of pain, migration to the right lower quadrant, and pain manifestation. For the remaining features, physician performance was slightly higher, with marginal accuracy differences. All feature-level outputs for this comparison are available in Multimedia Appendix 4.

### HIVE Model Performance Using LLM vs Physicians’ Inputs

**Figure 3.**
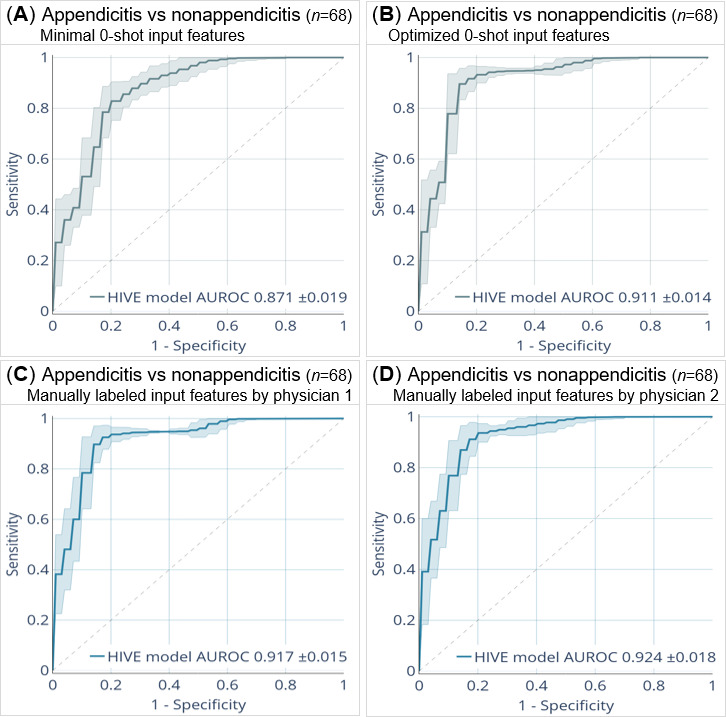
AUROC of the HIVE model for predicting appendicitis vs other AAP causes (ie, no appendicitis) in the validation set (n=68). (A,B) HIVE model performance using LLM-extracted features obtained through the minimal and optimized 0-shot prompting, respectively. (C,D) HIVE model performance using manually labeled features by ED physician 1 and physician 2, respectively. 95% CIs. AAP: acute abdominal pain; AUROC: area under the receiver operating characteristic curves; HIVE: History, Intake, Vitals, Examination; LLM: large language model.

To compare predictive performance using LLM- vs physician-labeled input features, the HIVE model was evaluated for its ability to distinguish appendicitis from other AAP causes on the validation set (n=68; Figure 3). Using LLM-extracted features, the HIVE model achieved an AUROC of 0.871 (95% CI ±0.019) with the minimal prompt, and 0.911 (95% CI ±0.014) with the optimized prompt. In comparison, AUROCs were 0.917 (95% CI ±0.015) using features labeled by physician 1, and 0.924 (95% CI ±0.018) using those from physician 2.

**Figure 4.**
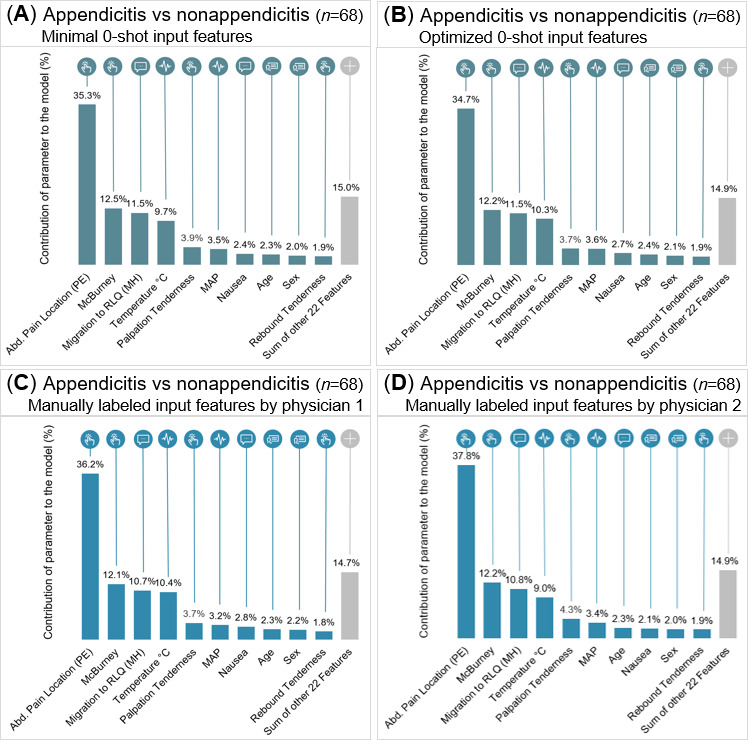
Feature contributions to the HIVE model across 4 different input types on the validation set (n=68). Contributions of each feature to the model are SHAP (Shapley Additive Explanations) values scaled and plotted as percentage contributions to the prediction. (A,B) Feature contributions to the HIVE model performance using LLM-extracted features obtained through the minimal and optimized 0-shot prompting, respectively. (C,D) Feature contributions to the HIVE model performance using manually labeled features by ED physician 1 and physician 2, respectively. Abd.: abdominal; ED: emergency department; HIVE: History, Intake, Vitals, Examination; LLM: large language model; MAP: mean arterial pressure; MH: medical history; PE: physical examination; RLQ: right lower quadrant.

SHAP analysis demonstrated consistent feature importance across all input types (Figure 4). Features are displayed in descending order of their mean absolute SHAP contributions. The overall SHAP distributions were nearly identical, with the top 10 features cumulatively contributing 85% to 85.9% of the model output (full list in Multimedia Appendix 5). Although the minimal 0-shot prompt achieved a lower AUROC of 0.871 (95% CI ±0.019), its feature importance pattern closely resembled those of the optimized and physician-labeled inputs.

The random forest performed similarly to the XGBoost-based HIVE model, with AUROCs of 0.909 (95% CI ±0.13) using the optimized prompt strategy and 0.883 (95% CI ±0.13) using the minimal prompt strategy (Multimedia Appendix 6).

### Error Analysis LLM

To examine misclassifications, 8 evaluation cases from the 4 lowest-performing features, pain manifestation, complaint development, pain onset, and abdominal-pain location, were reviewed to illustrate diverse error types.

**Figure 5.**
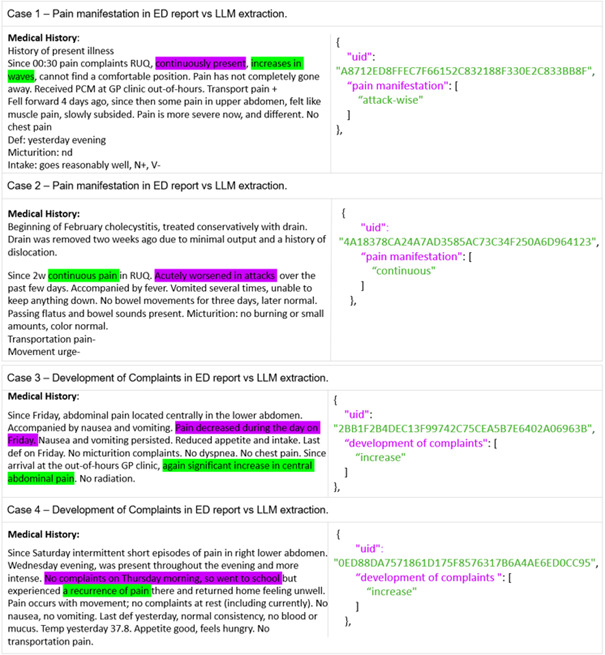
Case analysis of 8 ED reports across 4 features from the evaluation set. ED: emergency department; LLM: large language model.

**Figure 6.**
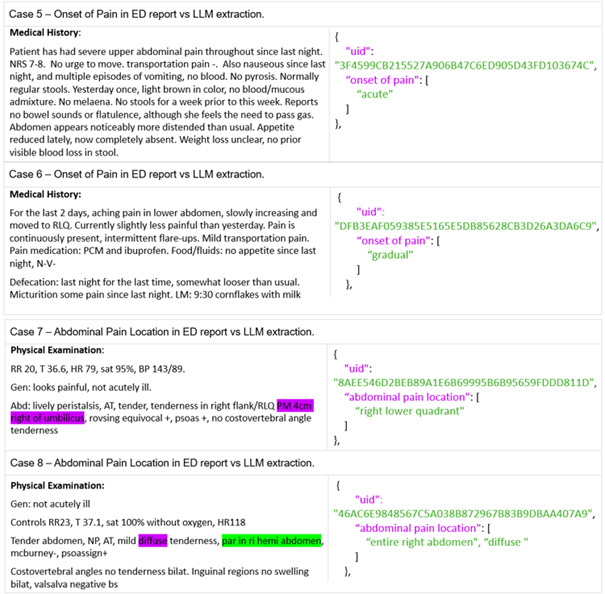
Case analysis of 8 ED reports across 4 features from the evaluation set. ED: emergency department; LLM: large language model.

The first 4 cases in Figure 5 highlight challenges with multilabel outputs, where the LLM was expected to report a combination of applicable labels but instead provided only a single answer. Specifically, in cases 1 and 2, pain manifestation evolved from continuous to attack-wise, yet the LLM recognized only 1 label. Similarly, in cases 3 and 4, the LLM missed fluctuating symptom development over time. In Figure 6, cases 5 and 6 illustrate 2 reports lacking explicit mention of the onset of pain; the LLM inferred labels such as “acute” or “gradual,” whereas reference was “not reported.” In case 7, the ED report described a punctum maximum located 4 cm to the right of the umbilicus. While this was labeled as “periumbilical” in the reference standard, the LLM returned “right lower quadrant.” In case 8, despite instructions to report only the primary pain location, the LLM provided both “entire right abdomen” and “diffuse” for a case describing diffuse abdominal pain with pronounced tenderness in the right hemiabdomen.

Each panel presents a snippet from an ED report alongside the corresponding LLM output. Words highlighted in magenta within the ED report indicate incorrect (false negative or false positive) or missed interpretations by the LLM, while words in green represent correct (true positive) interpretations. On the right-hand side, the LLM’s JSON output displays the unique patient identifier, the target clinical feature (magenta), and the extracted output (green). All prompts and ED reports were provided to the LLM in Dutch and have been translated into English for readability.

## Discussion

Conventional ML prediction models typically require structured input, posing a significant barrier to clinical integration, especially in EDs where physicians predominantly use free-text reports. This study demonstrates that a locally deployable LLM can automatically extract clinically relevant signs and symptoms from unstructured text, including binary, multiclass, and multilabel features. Using an optimized prompt strategy, the LLM reliably extracted features from free-text ED reports, enabling the appendicitis prediction model to achieve diagnostic performance comparable to that based on manually labeled features by ED physicians. This performance was consistent with that achieved in our previous study [18], underscoring the reliability of the LLM in this context.

Numerous ML prediction models have relied on clinical signs and symptoms to support early disease detection and guide ED workflows. While NLP techniques are commonly used to structure this data, each comes with limitations. Conventional techniques, such as bag-of-words, term frequency-inverse document frequency, and phrase skip-gram, rely on word frequency or co-occurrence, with limited ability to capture contextual meaning [32-36]. As a result, they often miss subtle language cues, such as symptom affirmation, negation, or uncertainty, which are essential in clinical interpretation. Topic modeling can uncover broad textual themes but may not align well with specific clinical concepts [33]. Moreover, these techniques are unsupervised, meaning they do not rely on labeled data, which makes their output more difficult to interpret and control. Deep learning models used in this context (eg, long short-term memory or BERT) understand the meaning of words in a broader context [37-40] but require fine-tuning to handle domain-specific language, large annotated datasets, and are challenged by imbalanced data distributions, limiting their performance and generalizability in real-world settings [41,42].

A small, locally hosted LLM for feature extraction can be deployed with modest requirements, offering advantages in data security and independence from external infrastructure. In comparison with conventional NLP or deep learning models, the LLM used in this study required neither extensive preprocessing nor fine-tuning nor large annotated datasets. Instead, it successfully extracted relevant features directly from raw text using relatively simple 0-shot prompting strategies. Importantly, the LLM was used solely for feature extraction; diagnostic predictions were generated by a separate ML prediction model. This modular approach preserves interpretability and statistical transparency, including calibrated probabilities and CIs. SHAP analysis confirmed that the model’s internal reasoning remained stable across input types: AUROC differences primarily reflected feature extraction accuracy rather than alterations in model reasoning. A random forest benchmark produced similar AUROCs to the XGBoost algorithm, indicating that the predictive signal in the LLM-extracted features is consistent across ML algorithms and not dependent on a specific model choice. Although the approach is lightweight and easy to deploy, real-world use still requires seamless EHR integration and fast inference. Modern EHR systems increasingly provide vendor APIs and Python-based toolkits for AI integration, but embedding such workflows into routine EHR practice remains a significant challenge [43]. Given the diagnostic complexity of appendicitis [44-46], this workflow could function as a safety net by flagging high-risk presentations and supporting diagnostic decisions. Larger multicenter evaluations, including physician performance with and without AI assistance, will be needed to determine real-world value.

The ablation study showed that adding specific, symptom-related context, including explanatory cues, domain-specific terminology, and negation or constraint handling, most consistently improved extraction performance. These elements enhanced the LLM’s ability to interpret synonyms and abbreviations commonly used in Dutch ED reports (eg, “misselijkheid,” “nausea,” or “N+” for nausea present) and to distinguish related but distinct concepts (eg, ignore vomiting when assessing nausea). In contrast, broader context elements such as report type, AI persona, or section limitation contributed little to performance. The LLM generalized well across features, classes, and prevalence levels, without requiring balancing strategies, and the close agreement with physician annotations suggests that more complex prompting strategies (eg, few-shot or chain-of-thought) add limited value for this task.

Despite its strengths, error analysis revealed that the LLM struggled with multilabel features, particularly when symptoms evolve over time. Clearly and consistently described features such as nausea or stool consistency were extracted more reliably than the onset of pain, which the LLM may have inferred from the surrounding context. In some cases, the LLM failed to prioritize the most prominent symptom, possibly due to reliance on textual similarity over clinical meaning, as observed in determining abdominal pain location. The LLM occasionally returned descriptive terms that matched the ED report but not the prescribed answer options. Some discrepancies also reflected inherent ambiguities between LLM output and manual annotations. Importantly, the LLM did not hallucinate nonexistent features outside the instructed features and answer options. These findings highlight the potential of LLM-based feature extraction, but also have limitations in handling time discrepancies or ambiguity, which are common challenges in clinical narratives.

This study has several limitations. Only 1 LLM (Qwen 2.5:14B) was evaluated, and performance may differ across models with different architectures or training corpora. As LLMs evolve, future LLMs with improved language understanding or instruction-tuned capabilities may outperform current results and reduce the need for prompt optimization. Nonetheless, given the complexity of ED reports, including abbreviations, medical jargon, and ambiguous phrasing, a structured prompting framework, as presented in this study, will likely remain necessary to ensure consistency and clinically accurate outputs. This study focused on Dutch-language ED reports, reflecting the clinical and linguistic context of our setting. While the prompting framework may be transferable, prompt content must be tailored to language-specific clinical conventions [47]. Translating Dutch reports would not serve as a valid proxy for cross-lingual evaluation because it measures translation quality rather than the LLM’s ability to process native clinical text. Although most open-source multilingual LLMs perform best in English, Chinese, and Spanish [17,48,49], the LLM in this study achieved strong results in Dutch, suggesting that carefully designed prompts can support effective use in underrepresented languages. Despite the single-center scope, the dataset spans 7 years and includes reports from many ED physicians with varying documentation styles, supporting robustness, but external validation across linguistic and clinical contexts is needed [50]. Finally, this study focused on extracting signs and symptoms, not numeric values as laboratory results or vital signs, which are typically stored in structured EHR fields.

This study demonstrates that a locally deployable LLM can automatically extract clinical signs and symptoms from free-text Dutch ED reports with performance comparable to manual labeling by two ED physicians using a structured prompt framework, even in a less widely represented language. This approach has the potential to support scalable implementation of our ML prediction models across clinical contexts, without extensive human annotation or computational resources. Ultimately, it may inform future development of real-time integration of prediction models into clinical workflows, facilitating more scalable, transparent, and privacy-preserving decision-support systems.

## Supplementary material

10.2196/81500 Multimedia Appendix 1 Development of structured features from free-text ED (emergency department) reports using researcher annotations.

10.2196/81500 Multimedia Appendix 2 Feature contributions to the HIVE (History, Intake, Vitals, Examination) model from the previous study’s validation set (n=68).

10.2196/81500 Multimedia Appendix 3 Elements evaluated in the prompt ablation study for each feature.

10.2196/81500 Multimedia Appendix 4 All outputs from the LLM (large language model) and ED (emergency department) physicians’ annotations (n=236).

10.2196/81500 Multimedia Appendix 5 Full list of feature contributions using SHAP (Shapley Additive Explanations) values scaled as percentage contributions to the HIVE (History, Intake, Vitals, Examination) model on the validation set (n=68).

10.2196/81500 Multimedia Appendix 6 AUROC (area under the receiver operating characteristic curves ) of the HIVE (History, Intake, Vitals, Examination) random forest model for predicting appendicitis vs other AAP (acute abdominal pain) causes in the validation set (n=68).

10.2196/81500 Checklist 1 TRIPOD Checklist.

## Abbreviations

AAP: acute abdominal pain
AI: artificial intelligence
AUROC: area under the receiver operating characteristic curve
BERT: Bidirectional Encoder Representations From Transformers
ED: emergency department
EHR: electronic health record
HIVE: History, Intake, Vitals, Examination
LLM: large language model
NLP: natural language processing
SHAP: Shapley Additive Explanations
TRIPOD: Transparent Reporting of a Multivariable Prediction Model for Individual Prognosis or Diagnosis
XGBoost: Extreme Gradient Boosting
