# Supplementary material for: Large Language Model Automated Extraction of Clinical Signs and Symptoms From Emergency Department Reports for Machine Learning Prediction Models: Development and Validation Study
Source: JMIR Med Inform. 2026 Apr 30;14:e81500. doi: 10.2196/81500 (PMC13136498; doi:10.2196/81500)
Supplement: Multimedia Appendix 1 — Development of structured features from free-text ED (emergency department) reports using researcher annotations. [file medinform-v14-e81500-s001.docx]

Medical history and physical examination data provided in free-text entries in the ED reports were extracted from ED reports for each case. To structure this data, an initial annotation process was conducted by two researchers, who labeled all medical symptoms in 100 cases, resulting in 367 initial labels. The annotations were performed using annotation software Doccano (version 1.4). Labels with a prevalence of less than 5% were then reviewed by two ED physicians for their diagnostic value. Those deemed clinically unrelated to AAP causes were excluded, while others were grouped under overarching labels, reducing the total to 289. This final set of 289 labels was categorized into 73 parameters. These parameters included 50 binary parameters (e.g., presence of nausea) and 23 nominal parameters (e.g., location of pain). Another 236 cases were subsequently annotated using this structured framework.
