# Supplementary material for: Large Language Model Automated Extraction of Clinical Signs and Symptoms From Emergency Department Reports for Machine Learning Prediction Models: Development and Validation Study
Source: JMIR Med Inform. 2026 Apr 30;14:e81500. doi: 10.2196/81500 (PMC13136498; doi:10.2196/81500)
Supplement: Multimedia Appendix 2 — Feature contributions to the HIVE (History, Intake, Vitals, Examination) model from the previous study’s validation set (n=68). [file medinform-v14-e81500-s002.docx]

**Table S1.** Feature contributions to the HIVE model from the previous study’s validation set ^a^ (*n*=68), excluding features with <0.01% contribution.

|  | **Data Categories** | **Features** | **% contribution to the HIVE model^b^** | **AUC** | **AUC CI (95%) Error Margin** |
| --- | --- | --- | --- | --- | --- |
| **1** | **Physical examination** | **McBurney's Sign** | **29.2%** | **0.75** | **0.059** |
| **2** | **Vital signs** | **Temperature [°C]** | **13.1%** | **0.822** | **0.035** |
| **3** | **Medical history** | **Pain Migration** | **12.5%** | **0.885** | **0.028** |
| **4** | **Vital signs** | **Mean Arterial Pressure [mmHg]** | **9.1%** | **0.904** | **0.018** |
| **5** | **Medical history** | **Nausea** | **4.6%** | **0.904** | **0.02** |
| **6** | **Vital signs** | **Oxygen Saturation [%]** | **4.0%** | **0.908** | **0.02** |
| **7** | **Vital signs** | **Heart Rate [bpm]** | **3.2%** | **0.906** | **0.025** |
| **8** | **Physical examination** | **Abdominal Pain Location** | **3.2%** | **0.916** | **0.023** |
| **9** | **Medical history** | **Fever** | **2.0%** | **0.918** | **0.02** |
| **10** | **Intake information** | **Referrer** | **1.7%** | **0.913** | **0.024** |
| **11** | **Intake information** | **Pain rating** | **1.6%** | **0.916** | **0.023** |
| **12** | **Medical history** | **Pain location** | **1.6%** | **0.917** | **0.023** |
| **13** | **Medical history** | **Pain manifestation** | **1.5%** | **0.917** | **0.021** |
| **14** | **Intake information** | **Sex** | **1.3%** | **0.917** | **0.023** |
| **15** | **Physical examination** | **Rebound tenderness** | **0.8%** | **0.913** | **0.021** |
| **16** | **Intake information** | **Age [y]** | **0.8%** | **0.917** | **0.023** |
| **17** | **Medical history** | **Anorexia** | **0.7%** | **0.92** | **0.02** |
| **18** | **Physical examination** | **Abdominal inspection** | **0.7%** | **0.919** | **0.023** |
| **19** | **Medical history** | **Stool consistency** | **0.6%** | **0.923** | **0.021** |
| **20** | **Medical history** | **Nature of pain** | **0.6%** | **0.925** | **0.02** |
| **21** | **Medical history** | **Onset of pain** | **0.6%** | **0.924** | **0.02** |
| **22** | **Intake information** | **Transport** | **0.5%** | **0.923** | **0.023** |
| **23** | **Vital signs** | **Diastolic blood pressure [mmHg]** | **0.4%** | **0.924** | **0.021** |
| **24** | **Medical history** | **Development of complaints** | **0.4%** | **0.923** | **0.021** |
| **25** | **Physical examination** | **Palpation tenderness (soft/supple)** | **0.4%** | **0.924** | **0.02** |
| **26** | **Vital signs** | **Q-SOFA** | **0.4%** | **0.926** | **0.022** |
| **27** | **Medical history** | **Pollakiuria^c^** | **0.4%^c^** | **0.929^c^** | **0.019^c^** |
| **28** | Medical history | Vomiting | 0.4% | 0.922 | 0.021 |
| **29** | Medical history | Food-related | 0.3% | 0.919 | 0.023 |
| **30** | Physical examination | Percussion pain | 0.3% | 0.92 | 0.022 |
| **31** | Medical history | Movement urge | 0.3% | 0.919 | 0.023 |
| **32** | Medical history | Last stool | 0.3% | 0.921 | 0.02 |
| **33** | Medical history | Transportation pain | 0.3% | 0.921 | 0.022 |
| **34** | Vital signs | SIRS | 0.2% | 0.921 | 0.02 |
| **35** | Physical examination | Palpation tenderness (pain on palpation) | 0.2% | 0.922 | 0.021 |
| **36** | Medical history | Duration complaints | 0.2% | 0.919 | 0.021 |
| **37** | Medical history | Medical history: prior appendectomy | 0.2% | 0.92 | 0.023 |
| **38** | Medical history | Menstrual abnormalities | 0.2% | 0.92 | 0.022 |
| **39** | Medical history | Sweatiness | 0.2% | 0.92 | 0.022 |
| **40** | Medical history | Surrounding people's complaints | 0.2% | 0.92 | 0.023 |
| **41** | Physical examination | Presence of abdominal auscultation | 0.2% | 0.92 | 0.022 |
| **42** | Medical history | Hematemesis | 0.2% | 0.92 | 0.023 |
| **43** | Medical history | Cold shivers | 0.2% | 0.919 | 0.023 |
| **44** | Vital signs | Systolic blood pressure [mmHg] | 0.2% | 0.919 | 0.021 |
| **45** | Medical history | Thoracic pain | 0.2% | 0.919 | 0.024 |
| **46** | Medical history | Swollen abdomen (MH) | 0.1% | 0.919 | 0.023 |

**^a^** Schipper A, Belgers P, O’Connor R, et al. Machine-learning based prediction of appendicitis for patients presenting with acute abdominal pain at the emergency department. World J Emerg Surg. 2024;19:40. PMID: 39716296. DOI: 10.1186/s13017-024-00570-7.

**^b^** % Contribution to the model are based on mean absolute SHAP values.

**^c^** Features up to and including pollakiuria were selected for this study and included in the new HIVE (prediction) model.
