# Supplementary material for: Large Language Model Automated Extraction of Clinical Signs and Symptoms From Emergency Department Reports for Machine Learning Prediction Models: Development and Validation Study
Source: JMIR Med Inform. 2026 Apr 30;14:e81500. doi: 10.2196/81500 (PMC13136498; doi:10.2196/81500)
Supplement: Multimedia Appendix 5 — Full list of feature contributions using SHAP (Shapley Additive Explanations) values scaled as percentage contributions to the HIVE (History, Intake, Vitals, Examination) model on the validation set (n=68). [file medinform-v14-e81500-s005.docx]

**Table S1.** full list of feature contributions using SHAP values scaled as percentage contributions to the HIVE model on the validation set (n=68).

| **Feature** | **Minimal Prompt** | **Optimized Prompt** | **ED physician 1** | **ED physician 2** |
| --- | --- | --- | --- | --- |
| **Abdominal Pain Location (PE)** | 35.3% | 34.7% | 36.2% | 37.8% |
| **McBurney's sign** | 12.5% | 12.2% | 12.1% | 12.2% |
| **Migration to RLQ** | 11.5% | 11.5% | 10.7% | 10.8% |
| **Temperature** | 9.7% | 10.3% | 10.4% | 9.0% |
| **Palpation Tenderness (supple)** | 3.9% | 3.7% | 3.2% | 4.3% |
| **Mean Arterial Pressure [mmHg]** | 3.5% | 3.6% | 3.7% | 3.4% |
| **Nausea** | 2.4% | 2.7% | 2.8% | 2.1% |
| **Age** | 2.3% | 2.4% | 2.3% | 2.3% |
| **Sex** | 2.0% | 2.1% | 2.2% | 2.0% |
| **Rebound Tenderness** | 1.9% | 1.9% | 1.8% | 1.9% |
| **Pain Rating** | 1.8% | 1.8% | 1.6% | 1.7% |
| **SIRS** | 1.5% | 1.6% | 1.6% | 1.2% |
| **Diastolic Arterial Pressure [mmHg]** | 1.4% | 1.5% | 1.5% | 1.3% |
| **Transport** | 1.4% | 1.2% | 1.3% | 1.5% |
| **Pain Location** | 1.2% | 1.1% | 1.1% | 1.0% |
| **Pain Manifestation** | 1.1% | 1.1% | 1.1% | 1.2% |
| **Heart Rate [bpm]** | 1.1% | 1.0% | 1.0% | 1.3% |
| **Development of Complaints** | 1.1% | 1.1% | 0.9% | 0.9% |
| **Nature of Pain** | 0.8% | 0.8% | 0.9% | 0.7% |
| **Prior ED visits with AAP** | 0.7% | 0.7% | 0.7% | 0.7% |
| **Pollakiuria** | 0.7% | 0.7% | 0.8% | 0.6% |
| **Systolic Arterial Pressure [mmHg]** | 0.6% | 0.6% | 0.6% | 0.5% |
| **Onset of Pain** | 0.5% | 0.5% | 0.5% | 0.5% |
| **Stool Consistency** | 0.4% | 0.5% | 0.5% | 0.4% |
| **Anorexia** | 0.4% | 0.4% | 0.4% | 0.4% |
| **Referrer** | 0.2% | 0.2% | 0.2% | 0.3% |
| **Abdominal Inspection** | 0.0% | 0.0% | 0.0% | 0.0% |
| **Respiratory Rate [rpm]** | 0.0% | 0.0% | 0.0% | 0.0% |
| **EMV** | 0.0% | 0.0% | 0.0% | 0.0% |
| **Fever** | 0.0% | 0.0% | 0.0% | 0.0% |
| **Q SOFA** | 0.0% | 0.0% | 0.0% | 0.0% |
| **Oxygen Saturation** | 0.0% | 0.0% | 0.0% | 0.0% |
