# Supplementary material for: Large Language Model Automated Extraction of Clinical Signs and Symptoms From Emergency Department Reports for Machine Learning Prediction Models: Development and Validation Study
Source: JMIR Med Inform. 2026 Apr 30;14:e81500. doi: 10.2196/81500 (PMC13136498; doi:10.2196/81500)
Supplement: Multimedia Appendix 6 — AUROC (area under the receiver operating characteristic curves ) of the HIVE (History, Intake, Vitals, Examination) random forest model for predicting appendicitis vs other AAP (acute abdominal pain) causes in the validation set (n=68). [file medinform-v14-e81500-s006.docx]

**Schipper, A., Belgers, P., O’Connor, et al. Large Language Model Automated Extraction of Clinical Signs and Symptoms from Emergency Department Reports for Machine Learning Prediction Models: A Development and Validation Study. 2025.**


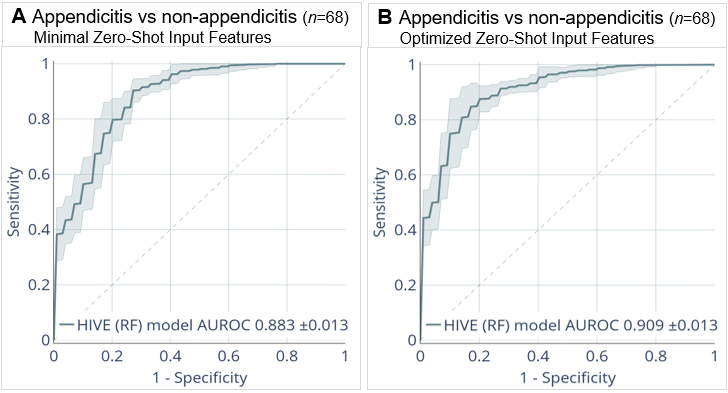


**Figure S1.** Area under the receiver operating characteristic curves (AUROC) of the HIVE Random Forest model for predicting appendicitis versus other AAP causes (i.e. no appendicitis) in the validation set (*n*=68).

**A**, **B** HIVE RF model performance using LLM-extracted features obtained through the minimal and optimized zero-shot prompting, respectively.

**Abbreviations:** *HIVE, History, Intake, Vital signs, physical Examination. AAP, acute abdominal pain. LLM, Large Language Model. RF, Random Forest.*
